# A comparative study on the reproductive success of two rewarding *Habenaria* species (Orchidaceae) occurring in roadside verge habitats

**DOI:** 10.1186/s12870-021-02968-w

**Published:** 2021-04-19

**Authors:** Wenliu Zhang, Jiangyun Gao

**Affiliations:** 1 Yunnan Key Laboratory of Plant Reproductive Adaption and Evolutionary Ecology, Yunnan University, Kunming, 650091 Yunnan China; 2 Laboratory of Ecology and Evolutionary Biology, Yunnan University, Kunming, 650091 Yunnan China

**Keywords:** Reproductive success, Plant density, Viable embryo, Geitonogamy, *Habenaria*

## Abstract

**Background:**

Most orchid species have been shown to be severely pollination limited, and the factors affecting reproductive success have been widely studied. However, the factors determining the reproductive success vary from species to species. *Habenaria* species typically produce nectar but exhibit variable fruit set and reproductive success among species. Here, we investigated the influence of the flowering plant density, inflorescence size, breeding system, and pollinator behaviour on the reproductive success of two rewarding *Habenaria* species.

**Results:**

Our observations indicated that *Habenaria limprichtii* and *H. petelotii* co-occur in roadside verge habitats and present overlapping flowering periods. Both species were pollination limited, although *H. limprichtii* produced more fruits than *H. petelotii* under natural conditions during the 3-year investigation. *H. petelotii* individuals formed distinct patches along roadsides, while nearly all *H. limprichtii* individuals clustered together. The bigger floral display and higher nectar sugar concentration in *H. limprichtii* resulted in increased attraction and visits from pollinators. Three species of effective moths pollinated for *H. limprichtii*, while *Thinopteryx delectans* (Geometridae) was the exclusive pollinator of *H. petelotii*. The percentage of viable seeds was significantly lower for hand geitonogamy than for hand cross-pollination in both species. However, *H. limprichtii* may often be geitonogamously pollinated based on the behaviours of the pollinators and viable embryo assessment.

**Conclusions:**

In anthropogenic interference habitats, the behaviours and abundance of pollinators influence the fruit set of the two studied species. The different pollinator assemblages in *H. limprichtii* can alleviate pollinator specificity and ensure reproductive success, whereas the more viable embryos of natural fruit seeds in *H. petelotii* suggested reducing geitonogamy by pollinators in the field. Our results indicate that a quantity-quality trade-off must occur between species with different breeding strategies so that they can fully exploit the existing given resources.

**Supplementary Information:**

The online version contains supplementary material available at 10.1186/s12870-021-02968-w.

## Background

The incredible diversity of orchids is widely attributed to adaptive radiation for specific pollinators driven by selection for out-crossing [1, 2]. Most orchid species use animal vectors to deliver pollen for sexual reproduction and have developed diverse mechanisms to promote pollination and increase reproductive success. In general, rewarding species secrete nectar that can be a substantial source of energy for pollinators and may encourage repeated visits by pollinators [3]; thus, rewarding orchids are more successful at producing fruits than their rewardless counterparts. In fact, the mean fruit set of rewarding orchids reached > 50% and was more than twice that of rewardless species in temperate and tropical areas [2, 4]. Fruit set varies among species, and numerous studies have investigated the factors affecting reproductive success in orchids, especially in deceptive species [2]. However, the factors that determine reproductive success vary from species to species.

Several factors have been found to influence the fruit set and pollination success of a rewarding orchid, and variations in population size, plant density, and inflorescence size may result in differences in attractiveness to pollinators (and thus in fruit set) among intraspecific or interspecific populations [5–7]. In general, these factors in rewarding plants can affect the behaviour of pollinators in two advantageous ways: creating highly conspicuous visual signals and increasing the number of flowers that pollinators can probe in a single visit [5]. A relatively larger population and higher plant density, which correspond to high number of flowering individuals, usually positively affect reproductive success because fruit set was suggested to be significantly higher in populations with more flowering individuals than in populations with fewer flowering individuals [8–11]. Moreover, increasing evidence indicates that inflorescence size is positively associated with the number of pollinator visits, resulting in increased fruit and seed production [12, 13].

In addition to the impact of the traits of orchids, the characteristics of pollinators, such as the pollinator abundance in a given habitat and pollinator visiting behaviours, are also main factors that affect reproductive success [14]. Since most rewarding species require pollinators to complete pollination, the effects of pollinators on plants can be reflected in the quantity and quality of pollen [2]. Pollen quantity, for example, may decrease as a result of a reduction in pollinator visits or a reduction in the pollen deposited per visit, whereas pollen quality can decrease if self-compatible or incompatible pollen is delivered [8, 15]. Fruit production in many orchids is pollen limited due to a scarcity of pollinators [2, 16], which may result in fluctuations in the rates of pollination and fertilization [14, 17, 18].

The reproductive success of orchids is generally quantified by the proportion of flowers that develop into fruits (fruit set). Several previous studies have discussed the reproductive success of orchids by providing fruit-set data [4, 12]; however, few studies have attempted to address the number of viable embryo seeds [6, 15]. Providing viable seed numbers in different treatments or in different species may provide further information on the productive success because highly viable embryos from seeds produced in fruit compensate for limited pollination and may ensure reproduction in populations [19]. High fruit set together with very high-quality seeds in orchid species may be considered the most effective reproductive strategy.

*Habenaria* Willd. is the largest primarily terrestrial orchid genus, and it includes approximately 880 species distributed throughout the tropical and subtropical regions of the Old and New World [20]. Much of the research related to pollination biology in *Habenaria* species is aimed towards a single species and focuses on pollinator diversity and the pollinia transfer efficiency in natural habitats [14, 15, 21]; however, previous studies have not considered the differences in the reproductive success between species occurring in anthropogenic interference habitats. Flowers in the genus often present spurs on the base filled with available nectar, and lepidopterans were the most commonly reported pollinators [14, 21–23]. As rewarding orchids, *Habenaria* species are usually considered high-fruit-set species, although the fruit set is still very low in some species [22–24]. Research on factors that may limit reproductive success between species is scarce.

Different *Habenaria* species often have sympatric distributions and overlapping flowering periods [22, 24]. *Habenaria petelotii* Gagnep. and *Habenaria limprichtii* Schltr. are both perennial terrestrial orchids that are widely distributed in southern and south-western China. *H. petelotii* occurs in a wide range with altitudes of 300–1600 m, while *H. limprichtii* is usually found in highlands at altitudes of 1900–3500 m [25]. In our field surveys on the diversity of orchid species in south-western Yunnan, we found the two species co-flowering and occurring in roadside verge habitats and determined that both had floral traits adapted to moth pollinators. A pervious study on two other sympatric *Habenaria* species in the same areas showed that the fruit set reached 80% over 3 years [26]. In this study, we studied the flowering plant density, pollinators, floral biology and breeding systems of *H. petelotii* and *H. limprichtii* to compare their reproductive success and identify the factors that may contribute to the differences between these species. Here, we present the results of our investigations, which addressed the following three principal questions concerning the reproductive biology of the two species: (1) What are the pollinators of *H. petelotii* and *H. limprichtii*? Do these plants share the same pollinators? (2) Is the natural fruit set different between the two rewarding *Habenaria* species? If so, do they have a similar proportion of viable seeds in natural fruits? (3) What are the possible factors that may affect the reproductive success of the two species?

## Results

### Population size and plant density

**Fig. 1:**
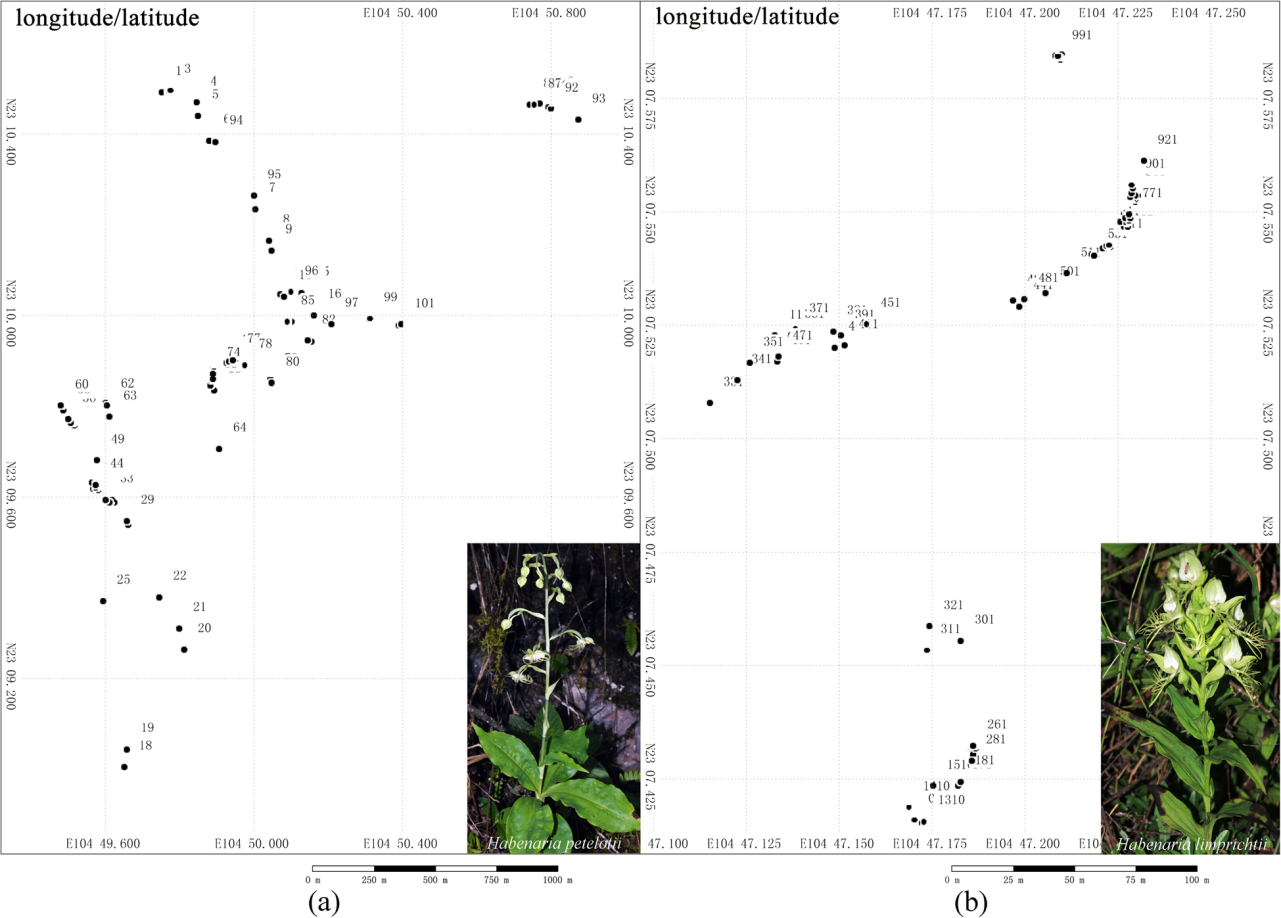
Population distribution patterns of *Habenaria petelotii* and *H. limprichtii* at the study sites in 2018. **a** Population distribution patterns of *H. petelotii* along the road near Daxiechang. **b** Population distribution patterns of *H. limprichtii* along the road near Shangchuandong

The two species had similar population sizes at the study site, and a total of 102 and 103 flowering plants of *Habenaria petelotii* and *H. limprichtii* were recorded and mapped in 2018, respectively (Fig. 1). *H. petelotii* individuals formed distinct patches along roadsides (Fig. 1a), while nearly all *H. limprichtii* individuals clustered together (Fig. 1b). The mean flowering plant number per patch (plant density) was 2.8 ± 0.47 for *H. petelotii* (range from 1 to 5 plants, *N* = 10 plots) and 11.2 ± 1.03 for *H. limprichtii* (range from 5 to 17 plants, *N* = 10 plots). As a result, *H. limprichtii* had a larger plant density than *H. petelotii* (*P* < 0.0001; Table 1).

**Table 1:** Floral traits and plant density of *H. petelotii* and *H. limprichtii* (mean ± SE). Statistically homogeneous groupings based on a one-way ANOVA

	*H. petelotii*	*H. limprichtii*	*F*	*P*
Flower length (mm)	29.93 ± 0.450 (*N* = 29)	42.36 ± 0.82 (*N* = 28)	185.826	*P <* 0.0001
Flower width (mm)	27.67 ± 0.36 (*N* = 29)	43.03 ± 1.09 (*N* = 28)	178.036	*P <* 0.0001
Spur length (mm)	16.37 ± 0.14 (*N* = 29)	19.61 ± 0.16 (*N* = 28)	233.372	*P <* 0.0001
Nectar volume (mm)	6.06 ± 0.34 (*N* = 29) (2.41–10.09)	4.88 ± 0.53 (*N* = 28) (2.37–15.56)	3.674	*P* = 0.06
Nectar sugar concentration (%)	14.80 ± 1.11 (*N* = 29) (13–19)	22.16 ± 0.67 (*N* = 28) (17–27.5)	32.271	*P <* 0.0001
Flowers per inflorescence	9.08 ± 0.48 (*N* = 133)	7.47 ± 0.24 (*N* = 196)	6.108	*P* = 0.0105
Floral longevity (days)	13.18 ± 0.35 (*N* = 60)	7.70 ± 0.14 (*N* = 54)	196.566	*P <* 0.0001
Plant density	2.8 ± 0.47 (*N* = 10) (1–5)	11.2 ± 1.03 (*N* = 10) (5–17)	55.125	*P <* 0.0001

### Flowering phenology and morphology

**Fig. 2:**
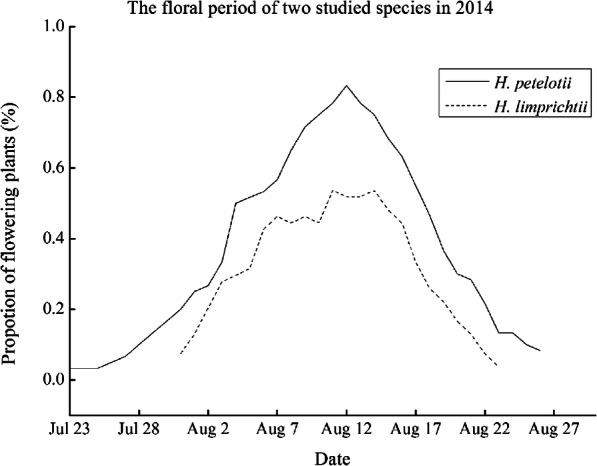
Flowering phenology (the proportion of flowering plants per day) of *Habenaria petelotii* and *H. limprichtii*. One census was taken per day from 23 July to 27 August 2014

**Fig. 3:**
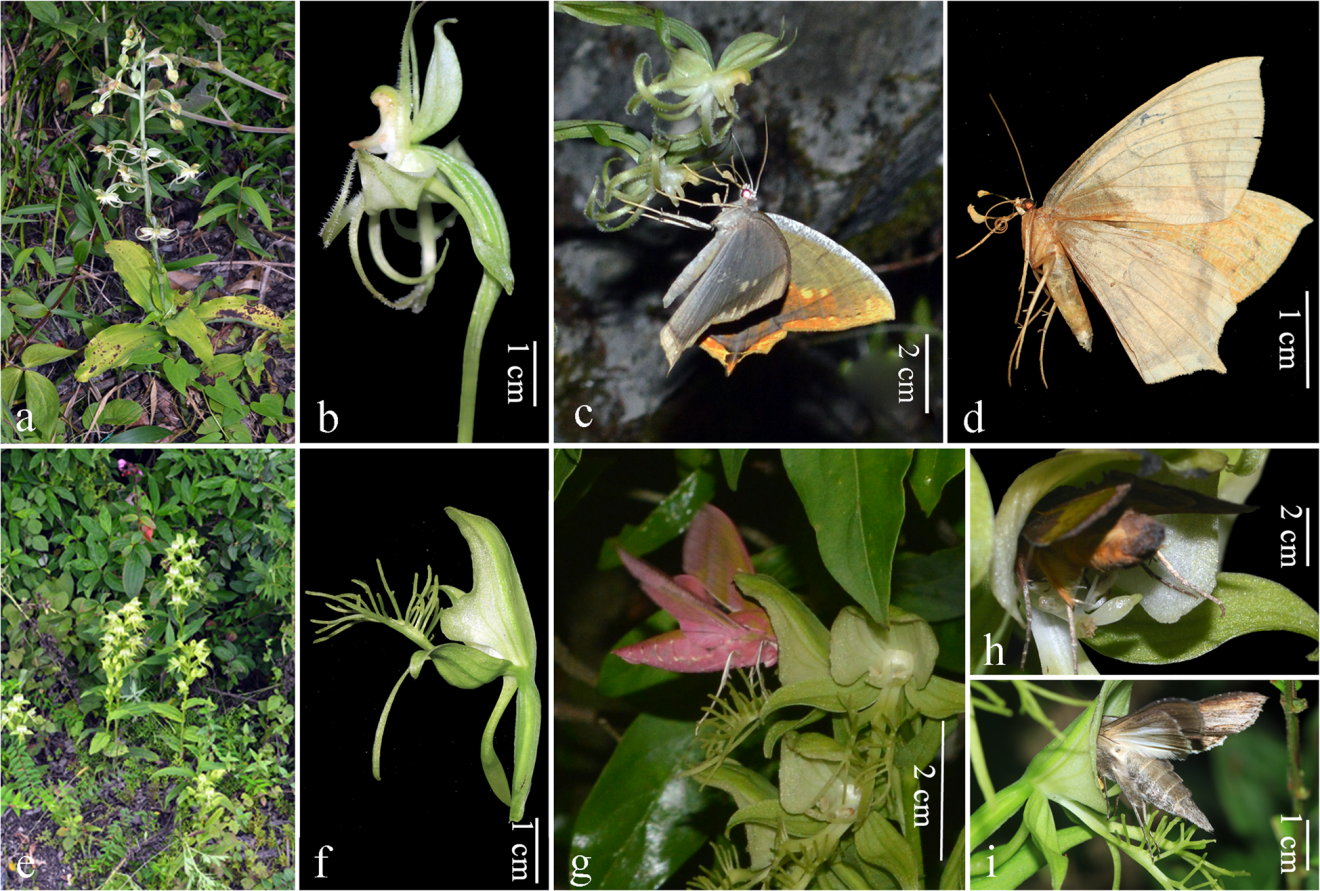
Plant, inflorescences, flowers and pollinators of *Habenaria petelotii* and *H. limprichtii*. **a** Plant and habitat of *H. petelotii*. **b** Single flower of *H. petelotii*. **c** *Thinopteryx delectans* visiting flowers of *H. petelotii* with pollinia attached to the base of its proboscis and legs. **d** *Thinopteryx delectans* with pollinia of *H. petelotii* attached to its head. **e** Plant and habitat of *H. limprichtii*. **f** Single flower of *H. limprichtii*. **g** *Deilephila elpenor* visiting flowers of *H. limprichtii*. **h** *Thysanoplusia intermixta* visiting flowers of *H. limprichtii.* **i** *Cucullia fraterna* visiting flowers of *H. limprichtii* with pollinia attached to the lateral-ventral side of its thorax

**Fig. 4:**
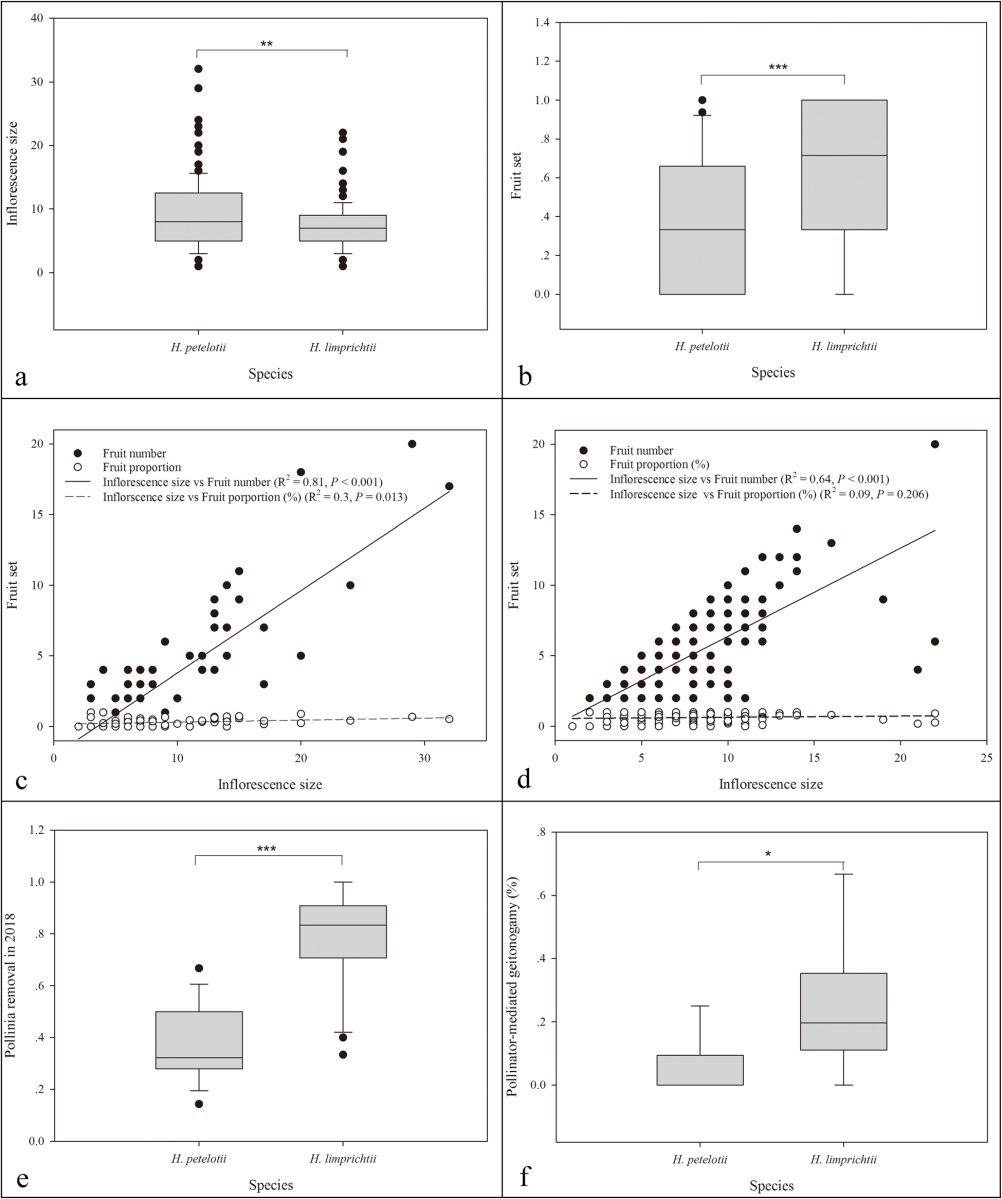
Comparisons between the two rewarding species, *Habenaria petelotii* and *H. limprichtii*. **a** Inflorescence size comparison between *H. limprichtii* and *H. petelotii.* **b** Overall natural fruit set during the 3 investigated years. **c** Correlation coefficient between the inflorescence size and fruit set in *H. petelotii*, and fruit set measured as the number of capsules produced (solid line) and proportion of fruits produced (dotted line). **d** The correlation coefficient between the inflorescence size and fruit set in *H. limprichtii*, and fruit set measured as the number of capsules produced (solid line) and proportion of fruits produced (dotted line). **e** Pollinia removal in 2018. **f** Pollinator-mediated geitonogamy

The two orchids flowered in August in our study site, and the flowering periods of the two species overlapped according to our observations in 2014, with *H. petelotii* flowering from 23 July to 26 August and *H. limprichtii* flowering from 31 July to 23 August (Fig. 2). Both species had 4–6 fleshy leaves on the ground and a racemose inflorescence at the top of the plants (Fig. 3a, e). Each individual of the two species produced a single inflorescence with 2–22 flowers, and the average number of flowers per inflorescence of *H. petelotii* was significantly greater than that of *H. limprichtii* (*P* < 0.001; Table 1 and Fig. 4a). The flowers of *H. petelotii* were loosely arranged on the inflorescences, while the flowers of *H. limprichtii* were tightly arranged, and all opened gradually from the bottom to the top (Fig. 3a, e). The *H. petelotii* flowers lasted 13.18 ± 0.35 days (*N* = 60), which was a significantly longer duration compared with that of the *H. limprichtii* flowers (7.70 ± 0.14 days, *N* = 54; *P* < 0.0001; Table 1).

The flowers of the two species were entirely greenish with white lateral sepals and a white labellum, and they were similar in structure. Each flower had two separate pollinia and stigmas, and the pollinia contained numerous massulae (Fig. 3b, f). The flower of *H. petelotii* was significantly smaller than the flower of *H. limprichtii* (length: *P* < 0.0001; width: *P* < 0.0001; Table 1). For both species, pendulous and cylindrical spurs were observed on the base of the flowers, and they were filled with nectar. The nectar volume and sugar concentration for each species varied markedly, with *H. limprichtii* presenting ca. 20% less nectar but with ca. 50% more sugar than *H. petelotii* (Table 1).

### Hand-pollination experiments and natural fruit sets

For the hand-pollination treatments in 2014, no fruit was found in the bagging and emasculation treatments. The fruit set did not differ between the geitonogamy and out-crossing treatments in *H. petelotii* (*P* = 0.073) and *H. limprichtii* (*P* = 0.164; Table 2). The natural fruit set of both species was significantly lower than the fruit set in the out-crossing treatments in 2014 (*H. petelotii*: *P* < 0.001; *H. limprichtii*: *P* = 0.006; Table 2), suggesting that fruit production was pollination limited in both species.

**Table 2:** Fruit set of the different hand-pollination treatments (bagging, emasculating, out-crossing, geitonogamy) in 2014, natural fruit set in 2014, 2015 and 2018, and viable embryo rate of seeds from geitonogamy, out-crossing and natural fruit in 2014 for the two *Habenaria* species (mean ± SE). The sample sizes of inflorescences and flowers are indicated in the brackets. Post-hoc multiple comparison tests (a, b, c) were conducted for the natural fruit set and viable seed embryos of *H. petelotii* and *H. limprichtii*

Treatments	Species	
	*H. petelotii*	*H. limprichtii*
Hand-pollination fruit sets % (inflorescences/flowers)		
Bagging	0 (5/31)	0 (5/42)
Emasculating	0 (5/22)	0 (5/29)
Out-crossing	92.22 ± 4.26 (15/51)	98.70 ± 1.30 (11/59)
Geitonogamy	66.87 ± 9.01 (18/85)	92.37 ± 2.63 (15/77)
Natural fruit sets % (inflorescences/flowers)		
2014	57.28 ± 7.50^a^ (31/250)	83.09 ± 3.55^a^ (40/322)
2015	30.16 ± 4.80^b^ (35/325)	68.12 ± 4.04^b^ (57/420)
2018	31.25 ± 3.47^b^(67/632)	51.27 ± 3.54^c^ (99/722)
Viable seed embryo rate % (fruits/seeds)		
Out-crossing	71.57 ± 1.85^a^ (10/2744)	58.23 ± 3.41^a^ (10/2076)
Geitonogamy	48.58 ± 2.57^c^ (10/2680)	13.16 ± 1.54^b^ (10/2377)
Natural fruit	58.99 ± 3.86^b^ (10/2530)	15.79 ± 3.26^b^ (10/2356)

The natural fruit set fluctuated in *H. petelotii* over 3 years and was significantly higher in 2014 than in the other 2 years (*P* < 0.001). Additionally, for *H. limprichtii*, the natural fruit set significantly differed among years, with the highest fruit set in 2014 and the lowest in 2018 (*P* < 0.001; Table 2). Overall, the natural fruit set in *H. limprichtii* was significantly higher than that in *H. petelotii* for the three investigated years (*P* < 0.001; Table 2 and Fig. 4b).

The two species revealed a clear and consistent pattern for the effect of the inflorescence size (number of flowers per inflorescence) on the number of fruit produced per individuals. The correlation coefficient between the inflorescence size and number of fruit produced was positive and significant in both *H. petelotii* (*R*^*2*^ = 0.81, *P* < 0.001; Fig. 4c) and *H. limprichtii* (*R*^*2*^ = 0.64, *P* < 0.001; Fig. 4d). However, the proportion of fruits produced per inflorescence was correlated with the inflorescences size in *H. petelotii* (*R*^*2*^ = 0.3, *P* = 0.013; Fig. 4c) but not in *H. limprichtii* (*R*^*2*^ = 0.09, *P* = 0.206; Fig. 4d).

For both species, the percentage of seeds with viable embryos was significantly higher in the out-crossing treatment than in the geitonogamy treatment and natural pollination (*H. petelotii*: *P* < 0.001; *H. limprichtii*: *P* < 0.001; Table 2). Moreover, the seed viability rate of geitonogamy treatment was higher in *H. petelotii* (48.58 ± 2.57%) than in *H. limprichtii* (13.16 ± 1.54%) (Table 2).

### Pollinia removal and deposition

Overall, pollinia removal and deposition were significantly lower in *H. petelotii* than in *H. limprichtii* (pollinia removal: *P* < 0.001; pollinia deposition: *P* < 0.001; Fig. 4e). The proportions of pollinia removal and deposition were 38.8 ± 2.31% and 53.66 ± 3.24% in *H. petelotii*, respectively, and 78.91 ± 2.99% and 81.27 ± 2.94% in *H. limprichtii*, respectively.

In the pollinia staining experiments, the proportion of labelled pollinia removal was 38.39 ± 7.22% in *H. petelotii* and 67.44 ± 9.09% in *H. limprichtii*, and the percent of stigmas that received pollinia from the same inflorescence was significantly higher in *H. limprichtii* (24.28 ± 7.35% of flowers; 60 flowers/8 inflorescences) than in *H. petelotii* (4.69 ± 3.29%; 64 flowers/8 inflorescences) (*P* = 0.02; Fig. 4f).

### Floral visitor observations

For all inflorescences bagged only during the observation time, no pollinia removal or deposition had occurred, indicating that insects had not visited the species out of our observation time.

Visitor observations was performed for a total of 90 h for *H. petelotii* from 2014 to 2016 in the field, and only one species of moth, *Thinopteryx delectans* (Geometridae), was observed and identified as a pollinator of *H. petelotii* (Fig. 3c, d). When visiting flowers of *H. petelotii*, the moth grasped the labellum by its forelegs and inserted its proboscis into the spur. It spent 30–40 s (*N* = 6) on one flower and visited 1–2 flowers (*N* = 5) per visitation. This moth usually carried more than three pollinia on its legs and at the base of its proboscis (Fig. 3c, d). We recorded ten visits in 2014 and three visits in 2016 but failed to observe any visitors in 2015, which was probably because of the poor weather conditions during our observation period (Table 3).

**Table 3:** Total observation hours, pollinators, pollinator proboscis length, pollinator visiting times observed and the body parts that pollinarium attached for *H. petelotii* and *H. limprichti*

Species	TOH^a^	Pollinators	PL^b^ (mm)	TVT^c^	Body parts pollinia attached
*H. petelotii*	90 h	*Thinopteryx delectans* (Geometridae)	17.49 ± 1.12 (*N* = 2)	7	the base of proboscis & legs
*H. limprichtii*	62 h	*Deilephila elpenor* (Sphingidae)	27.52 ± 1.05 (*N* = 3)	3	eyes
		*Thysanoplusia intermixta* (Noctuidae)	18.01 ± 0.64 (*N* = 8)	23	lateral-ventral side of thorax
		*Cucullia fraterna* (Noctuidae)	21.86 ± 1.04 (*N* = 5)	6	lateral-ventral side of thorax

^a^*TOH* Total observation hours, ^b^*PL* Roboscis length, ^c^*TVT* Total visiting times

For *H. limprichtii*, three species of effective pollinators were observed and recorded during the 62 h observation period in 2014 and 2015 (Table 3; Fig. 3g–i). *Deilephila elpenor* was observed and recorded visiting and pollinating flowers in both studied years. When it hovered at the front of the flowers and inserted its proboscis into the spur, its head pressed against the floral column, and pollinia adhered to its head (Fig. 3g). This hawkmoth usually spent 4–6 s (*N* = 11) on one flower and visited 3–4 flowers of one inflorescence (*N* = 6) per visitation. *Thysanoplusia intermixta* (Noctuidae) was observed only in 2014, and it had a high visiting frequency to flowers of *H. limprichtii*, with 4–6 visiting times and 2–4 individuals visiting every observation night from 17:00 to 21:00 (Fig. 3h). This moth moved frequently among inflorescences and spent approximately 25 s on one flower. The visiting behaviours of *Cucullia fraterna* (Noctuidae) were similar to those of *T. intermixta* on *H. limprichtii* flowers, and the pollinia of *H. limprichtii* were attached to its thorax (Fig. 3i). The proboscis length of the three pollinators was all well matched with the spur length of *H. limprichtii* (Table 3).

For the light trap experiment, two *D. elpenor* individuals were trapped and pollinarium was observed on the eyes of each individual; however, the other two pollinators of *H. limprichtii* were unavailable. The only pollinator of *H. petelotii* was not captured over the two continuous study days in 2018.

## Discussion

*Habenaria* species are characterized by the presence of floral spurs, the production of sucrose-rich nectar, and the emission of sweet scent during flowering that attract lepidopteran pollinators. The most common pollinators of *Habenaria* spp. are settling moths and hawkmoths based on the published research reports [22–24, 27]. Although most orchid species have been reported to be severely pollination-limited [2], previous studies indicated that *Habenaria* species usually showed various percentages of pollinia reception or fruit set (ranging from 3.82 to 86.1%) [22, 27].

The lack of fruit production in the bagging and emasculation treatments indicated that both studied species needed insects to achieve pollination and that spontaneous autogamy and apomixes did not occur. Natural fruit set was significantly lower than the fruit set from the hand geitonogamy and hand out-crossing treatments, suggesting that the fruit production of each species under natural conditions was both pollinator- and resource-limited. Overall, *H. limprichtii* had a higher natural fruit set and pollinia removal than *H. petelotii* (Fig. 4b, e)*.* Plant density can influence population dynamics such that the effect of pollen limitation is reduced in species with high density populations [10, 11, 28]. In this study, the difference in natural fruit set between *H. petelotii* and *H. limprichtii* may have been influenced by the difference in plant density, with *H. limprichtii* presenting a greater plant density than *H. petelotii* (< 0.0001; Table 1). *H. petelotii* usually grows sparsely with small groups of individuals separated by large distances (Fig. 1a), whereas *H. limprichtii* individuals consistently grow in large and dense populations (Fig. 1b). Therefore, within a certain range, the number of flowering *H. limprichtii* individuals is always greater than the number of flowering *H. petelotii* individuals, resulting in higher attractiveness to pollinators. Increases in plant density are likely to increase the visual signals and affect pollinator foraging behaviour by attracting more pollinators and reducing travel times between flowers, thus enhancing the pollinator foraging efficiency. Moreover, more flowers tend to attract more pollinators since they provide more floral resources (nectar, pollen, etc.). Therefore, flowers in dense patches usually produce higher fruit sets than flowers in sparse patches [10, 29]. Our pollinator observations confirmed that the flower of *H. limprichtii* received more pollinator visits than that of *H. petelotii.*

Previous studies on orchid species showed that the inflorescence size (number of flowers per inflorescence) is one of the factors that affect the reproductive success, although fruit set does not always increase linearly with inflorescence size [2]. Numerous positive cases [5, 12, 30, 31] and negative/uncorrelated cases [32–34] have been observed in orchid species. For the studied species, our results showed a positive correlation between the number of flowers developing into fruit and the number of flowers per inflorescence (Fig. 4c, d). However, the proportion of fruit produced per inflorescence was correlated with the inflorescence size in *H. petelotii* but not in *H. limprichtii*. This finding indicated that the increased number of fruits was not proportional to the increased size of the inflorescence in *H. limprichtii*. Therefore, compared with *H. petelotii*, the higher natural fruit set and greater pollinator attraction in *H. limprichtii* represented benefits of the higher flower and plant density but not the floral display of an individual plant.

In addition to the characteristics influencing pollination success in plants, fruit production in many orchids is also pollen limited due to a scarcity of pollinators [2, 16], which may result in fluctuations in the rates of pollination and fertilization [17, 18]. Xiong et al. [14] recorded pollinia movement over 8 years in *H. glaucifolia* and found that failure to receive pollinia was due to a scarcity of hawkmoth pollinators. Our results indicated that the dominant pollinator of *H. limprichtii* (*D. elpenor*) was abundant in the study area in 2018. *D. elpenor* is a widely distributed hawkmoth and a highly efficient pollinator of *H. limprichtii*, which is consistent with the results of Tao et al. [15]. *H. petelotii* only had one pollinator, suggesting a specialist pollination system in this species, whereas *H. limprichtii* had three effective pollinators in the observed years, suggesting a generalized pollination system in anthropogenic interference habitats, roads generally have a negative effect on the occurrence and diversity of insects, with traffic noise affecting the behaviour of insects; thus, they often avoid roads [35]. Different pollinator assemblages can alleviate pollinator specificity and ensure reproductive success, particularly when the abundance of pollinators fluctuates among years [36]. This finding may explain why the natural fruit set of *H. limprichtii* was higher than that of *H. petelotii* in each studied year. Limited pollinator visitation could arise if pollinators are rare in the environment and/or if plants fail to attract available pollinators. *H. petelotii* seemed susceptible to both problems.

The topographical tetrazolium (TTC) test [37] has been used frequently as a direct method to determine orchid seed viability, and it has been used successfully on tropical epiphytic orchid seeds [38, 39] and adapted for several terrestrial species [40–42]. The number of viable seeds determined by the TTC test in our studies was significantly higher in the out-crossing treatment than in the geitonogamy treatment and natural pollination for both species. Generally, highly deleterious (often recessive) mutations are maintained in a given population of out-crossing species, as they are masked by heterozygosis. Therefore, manually selfing any plant that usually out-crosses will force the homozygosis of recessive deleterious mutations, and thus, we expect to observe relatively more strongly deleterious variants and a reduction in fitness of the progeny [43]. The significantly lower seed viability for hand geitonogamous pollination indicates that the populations of the two species are both predominantly out-crossed with high heterozygosity. However, different percentages of viable seeds in our geitonogamy treatments suggested there is a different level of homozygosis in the natural populations of the two species. The smaller number of viable embryos of natural fruit seeds in *H. limprichtii* suggests that high fruit set mediated by pollinators does not lead to higher production of viable seeds in the field. According to the pollinator visit behaviour of *H. limprichtii*, i.e., *D. elpenor* visit several flowers on the same inflorescence or return to the same inflorescence during the same foraging bouts, and the possibility of geitonogamy assessed by staining pollinia (Fig. 4f), the results suggest that geitonogamy by pollinators is a main factor for the decrease in viable natural fruit seeds in *H. limprichtii*. Other studies on orchids, also suggested that part of the observed fruit set is a consequence of pollinator-mediated geitonogamy based on observing their pollinators [15, 22]. In general, our results speculate that the *H. limprichtii* population may present high risk of inbreeding depression by pollinator-mediated geitonogamy. Additionally, pollination in low genetic diversity clusters may also decrease the number of viable embryos in natural fruits. The genetic relatedness of the individuals in these clusters formed by *H. limprichtii* must be determined in future research.

In orchids, different species usually have varied breeding strategies to ensure sexual reproduction. For instance, rewarding and deceptive pollination are alternative solutions that can provide a trade-off between pollination quality and quantity because deceptive species usually have low fruit set but produce more out-crossing seeds [44]. Similarly, for the rewarding species, the population distribution pattern and pollinator abundance determine the difference in pollination frequency and fruit set among species [2, 4]. Long-distance foraging by moths is expected to result in higher frequencies of cross-pollinated individuals [45, 46]. In this study, *H. petelotii* had low fruit set but produced more viable seeds in the field; conversely, *H. limprichtii* showed a high level of natural fruit set mediated by pollinators but resulted to a low proportion of viable seed. These results indicated that a quantity-quality trade-off must occur between species with different breeding strategies so that the existing given resources can be fully exploited.

## Conclusions

*Habenaria* species typically produce nectar but exhibit variable fruit set and reproductive success among species. Our results indicated that both species were pollination limited, although *H. limprichtii* produced more fruits than *H. petelotii* under natural conditions during the 3-year investigation. These two species had distinct plant densities and pollinator assemblages that together may contribute to differences in fruit production. However, the assessment of embryo viability indicated that each species showed different levels of embryo lethality after manual selfing, suggesting different rates of inbreeding/outbreeding of the natural populations. Genetic information for the studied populations is necessary to conclude on these aspects. The results suggested that a quantity-quality trade-off must occur between species with different breeding strategies so that the existing given resources can be fully exploited.

## Methods

### Studied species and study sites

Both *Habenaria petelotii* and *H. limprichtii* are terrestrial orchids, and they usually flower in August or September and have mature fruits in October. After the seeds are fully mature, the above-ground parts are completely withered, with only the tubers maintaining vitality. New leaves usually emerge in the following spring. The formal identification of plant materials was undertaken by the first author of this article (Dr. Wenliu Zhang). The herbarium vouchers of *H. petelotii* (No. 02069204) and *H. limprichtii* (No. 02069205) were deposited in the Herbarium of School of Life Science, Yunnan University.

This study was conducted along the roadsides from Daxiechang village (23°09′N, 104°50′E; alt. 1508 m) to Shangcuandong village (23°08′N, 104°47′E; alt. 2120 m) in Malipo County, south-eastern Yunnan Province, China. The two studied species grow near each other, with *H. petelotii* occurring in the lowest altitude range of 1340–1780 m and *H. limprichtii* occurring in the highest altitude range of 1972–2010 m. Two other species, *H. fordii*, with an altitude range of 1508–1800 m and *H. davidii*, with an altitude range of 1760–2120 m, overlapped in the distribution range [26]. At the study site, the two studied orchid species grew in crevices of calcareous rocks or in thickets along roadsides.

No specific permits were required for the described field studies, because endangered or protected species were not involved, and the localities involved are not privately owned or protected in any way.

### Surveys on the population size and plant density

At the study site, all flowering individuals of *H. petelotii* and *H. limprichtii* were investigated, and the spatial distribution of all plants was mapped using a high-precision GPS (Garmin International Inc., KS, USA) in the flowering season of 2018. The plant density was measured according to a previously described method [47]. We randomly selected and marked ten flowering individuals as focal plants for each species and then determined the number of flowering individuals within a radius of 5.0 m around the focal plants. The number of flowering individuals around a focal plant was taken as one sample, and the average number of ten samples was calculated as the plant density.

### Flowering phenology and morphology

To compare the flowering phenology between *H. petelotii* and *H. limprichtii*, 100 marking individuals were observed and recorded during the flowering seasons. Daily, the number of flowering inflorescences, the number of flowers on the inflorescences, the flower arrangement on the inflorescences, and the total number of open flowers per inflorescence as well as the floral longevity at the end of the floral season were recorded. The flowering period differences between the two species were determined by calculating the proportion of flowering plants per day for each species.

At least 28 newly opened flowers from different individuals of each species were randomly selected to measure the size of flowers and flower parts using the same methods described by Zhang and Gao [26]. The sample size and the measured floral characteristics were detailed in Table 1.

### Hand-pollination experiments and natural fruit sets

To examine the breeding systems of the two species, different hand-pollination treatments were conducted at the study site in 2014 following the methods used in our previous study [26], including bagging treatment, emasculation treatment (pollinia were carefully removed 1 day before anthesis), geitonogamy treatment (flowers were hand-pollinated with pollinia from the same individual) and out-crossing treatment (flowers were hand-pollinated with pollinia of other individuals at least 100 m away). All the sampled individuals were bagged with nylon mesh before flower opening, bagged again after hand-pollination, and removed once the experiments were finished.

The natural fruit set of the two species was investigated by randomly marking inflorescences from different individuals in 3 years (2014, 2015 and 2018). To assess the correlation between the inflorescence size (measured as the number of flowers) and fruit set (measured as the number of capsules produced/proportion of fruits produced), we marked 67 individuals of *H. petelotii* and 99 individuals of *H. limprichtii* in 2018 and counted the flowers and capsules on each individual/proportion of fruits per inflorescence for subsequent analysis. The fruit set of the four hand-pollination treatments and the natural fruit set for each species were counted approximately 6 weeks later in the middle of October. The numbers of flowers and individuals are presented in Table 2.

The seed viability was compared by examining the percentage of seed with viable embryos in the fruits from the hand geitonogamy, cross-pollination and natural pollination treatments between the two species in 2014. At least 20 mature and indehiscent fruits from each treatment of the two species were harvested, and the fruits were dried using the same methods for long-term storage described by Zi et al. [48]. A subset of seeds was tested for viability using the TTC test described by Yeung et al. [49]. Seeds were examined under a stereomicroscope (DM 3000, Leica, Germany) and assessed as viable (pink or red embryo) or unviable (unstained embryo) (Fig. S1). Ten fruits replicates were taken from each treatment (geitonogamy, out-crossing and natural pollination) of *H. petelotii* and *H. limprichtii*.

### Pollinia removal and deposition

To estimate pollination success, 30 individuals of *H. petelotii* and 32 individuals of *H. limprichtii* were marked before anthesis in 2018, and pollinia removal and deposition were monitored and recorded daily. After anthesis, the proportion of pollinia removal and deposition was calculated as the number of flowers with pollinia removed and deposited divided by the total number of flowers examined.

To assess the degree of geitonogamous pollination caused by pollinators under natural conditions, eight continuously distributed inflorescences with six to ten fresh and unpollinated flowers of each species were selected and marked at the studied sites on August 16, 2018. The pollinia on marked flowers were labelled with histochemical stains (Fuzheng Donghai Food Co., Ltd., China) by using the method of Peakall [50] on the same day. To stain the pollinia, 2 μl of stain was carefully injected into the anther flaps, which supports the pollinia lobes, by using a 10 μl syringe. All pollinia on an inflorescence were labelled with one colour stain, and different colour stains were applied to different inflorescences. Based on the stain colour, marked pollinia deposited on the stigmas were monitored and recorded daily, and the percentage of flowers pollinated geitonogamously was calculated at the end of anthesis.

### Floral visitor observations

The behaviours of floral visitors for each species were observed during three continuous flowering seasons from 2014. The observations were mainly conducted from 18:00 to 23:00 local time because our initial survey in 2013 indicated that no diurnal visitors were observed visiting the two orchids. A small flashlight covered with a thick red plastic film was used to observe floral foragers at night [51]. To determine whether there are any pollinators visiting outside the observation period, we bagged some randomly selected inflorescences only during the observation period and monitored the flowers twice each day to determine whether there was any removal or deposition of the pollinia.

The total observation hours spent on each species are detailed in Table 3. We observed and recorded the behaviours of the flower visitors in detail, which included the time and frequency of visitation, the number of flowers/inflorescences per visitation, the number of flowers visited per inflorescence and the visiting time spend on a single flower. To identify the species and measure the morphology, we attempted to capture five pollinator individuals at the end of the observation period.

To understand the richness of different pollinators of two species in the study site, a patch with more than ten flowering individuals was selected for each species. Hawkmoths and moths were collected using light traps from 19:00–22:00 on August 19 and 24, 2018. The frequency of pollinator collection and the pollinarium number carried by the pollinators were investigated and recorded.

### Data collection and statistical analysis

To test whether measures of reproductive success increased with the number of flowers per inflorescence, the relationship between the inflorescence size and fruit set was assessed for each species in 2018 using linear or nonlinear regression.

The plant density, floral traits, pollinia removal and deposition percentage, and fruit set viable seed percentage of the two studied species were statistically compared. The homogeneity of variance was tested before the analysis (Table S1). For equal variance (*P* > 0.001), a one-way ANOVA was conducted; otherwise, a nonparametric test of the Mann-Whitney U-test was conducted. All statistical analyses were performed in SPSS ver. 22.0 for Windows (SPSS Inc., 14 Chicago, IL, USA).

## Supplementary Information

**Additional file 1: Figure S1.** The viable/non.viable seeds of *H. petelotii* (a) and *H. limprichtii* (b) tested by TTC method. Red arrows show the viable seeds with stained embryos. Black arrows show the non.viable seed that embryo development started but seeds were non-viable.

**Additional file 2: Table S1.** Statistical analyses between different groups. The homogeneity of variance was tested before the analysis. For equal variance, a one-way ANOVA (*P* > 0.001); otherwise, the nonparametric Mann-Whitney U test was conducted. The F value is shown for the one-way ANOVA test, and the Z value is shown for the Mann-Whitney U test.

## Data Availability

The datasets used and/or analysed during the current study are available from the corresponding author on reasonable request.
