# Supplementary material for: A comparative study on the reproductive success of two rewarding Habenaria species (Orchidaceae) occurring in roadside verge habitats
Source: BMC Plant Biol. 2021 Apr 19;21:187. doi: 10.1186/s12870-021-02968-w (PMC8054408; doi:10.1186/s12870-021-02968-w)

Fig. S1 The viable/non.viable seeds of *H. petelotii* (a) and *H. limprichtii* (b) tested by TTC method. Red arrows show the viable seeds with stained embryos. Black arrows show the non.viable seed that embryo development started but seeds were non-viable

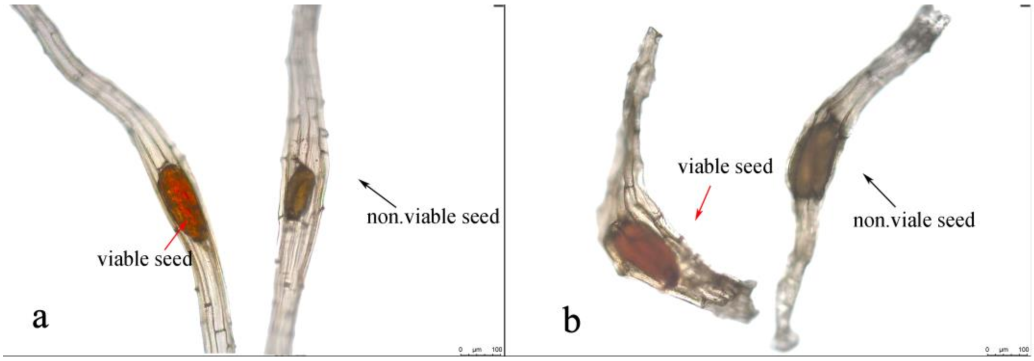

Supplement: Supplementary file 1 — Additional file 1: Figure S1. The viable/non.viable seeds of H. petelotii (a) and H. limprichtii (b) tested by TTC method. Red arrows show the viable seeds with stained embryos. Black arrows show the non.viable seed that embryo development started but seeds were non-viable. [file 12870_2021_2968_MOESM1_ESM.pdf]
