# Supplementary material for: A comparative study on the reproductive success of two rewarding Habenaria species (Orchidaceae) occurring in roadside verge habitats
Source: BMC Plant Biol. 2021 Apr 19;21:187. doi: 10.1186/s12870-021-02968-w (PMC8054408; doi:10.1186/s12870-021-02968-w)
Supplement: Supplementary file 2 — Additional file 2: Table S1. Statistical analyses between different groups. The homogeneity of variance was tested before the analysis. For equal variance, a one-way ANOVA (P > 0.001); otherwise, the nonparametric Mann-Whitney U test was conducted. The F value is shown for the one-way ANOVA test, and the Z value is shown for the Mann-Whitney U test. [file 12870_2021_2968_MOESM2_ESM.pdf]

Table S1. Statistical analyses between different groups. The homogeneity of variance was tested before the analysis. For equal variance, a one-way ANOVA (*P* > 0.001); otherwise, the nonparametric Mann-Whitney U test was conducted. The F value is shown for the one-way ANOVA test, and the Z value is shown for the Mann-Whitney U test

	Statistical Group	F/Z	*P*
Habenaria. petelotii	Fruit set: geitonogamy *vs* out-crossing	Z = 2.098	*P* = 0.073
	Fruit set: natural *vs* out-crossing	Z = -3.227	*P* < 0.001
	Natural fruit set: 2014 *vs* 2015 *vs* 2018	Z = 10.027	*P* = 0.007
	Seed viability: geitonogamy *vs* out-crossing *vs* natural	F = 15.947	*P* < 0.001
Habenaria limprichtii	Geitonogamy *vs* out-crossing	Z = 1.828	*P* = 0.164
	Natural *vs* out-crossing	Z = -2.723	*P* = 0.006
	Natural fruit sets: 2014 *vs* 2015 *vs* 2018	Z = 27.444	*P* < 0.001
	Seed viability: geitonogamy *vs* out-crossing *vs* natural	F = 77.769	*P* < 0.001
H. petelotii vs. H. limprichtii	Natural fruit set	F = 45.481	*P* < 0.001
	Seed viability of geitonogamy	F = 129.807	*P* < 0.001
	Pollinia removal	F = 113.906	*P* < 0.001
	Pollinia receipt	F = 39.958	*P* < 0.001
	Labelled pollinia removal	F = 6.257	*P* = 0.025
	Labelled pollinia deposition (natural geitonogamy)	F = 5.919	*P* = .029
